# Oral health promotion in adults with type 2 diabetes–a scoping review

**DOI:** 10.3389/fmed.2026.1852350

**Published:** 2026-07-29

**Authors:** Yahya Alholimie, Diaa Almutairi, Esraa Nassir Almarzooq, Jafar Alabdullah, Ola B. Al-Batayneh

**Affiliations:** 1 Dental Department, Prince Saud Bin Jalawy Hospital, Al Mubarraz, Saudi Arabia; 2 Dental and Oral Health Department, Prince Sultan Military College of Health Sciences, Dhahran, Saudi Arabia; 3 Dental Department, King Faisal General Hospital, Al Hofuf, Saudi Arabia; 4 University of Doha for Science & Technology, Doha, Qatar; 5 Jordan University of Science and Technology, Irbid, Jordan

**Keywords:** access to dental care, oral health promotion, patient education, periodontal disease, scoping review, type 2 diabetes mellitus

## Abstract

**Objective:**

Individuals affected with type 2 diabetes mellitus (T2DM) are always at higher risk for developing oral diseases. The current scoping review was undertaken to map the extent, range and nature of the recent evidence on oral-health promotion and prevention in adults with T2DM, focusing on patient awareness, provider practices, barriers to care, and preventive strategies.

**Methods:**

The review followed the Arksey and O'Malley framework as refined by Levac et al. and is reported in line with the PRISMA extension for Scoping Reviews (PRISMA-ScR). Five databases (PubMed, MEDLINE, Web of Science, ProQuest and Google Scholar) were searched for peer-reviewed studies published in English between 2015 and 2025. Records were screened independently by two reviewers against pre-defined eligibility criteria; data were charted and synthesized narratively into themes aligned to the research questions.

**Results:**

Of 412 records identified, nine peer-reviewed studies met the inclusion criteria. Three themes were identified: (i) associations between oral-hygiene practices, glycemic control and oral-health outcomes; (ii) patient knowledge and awareness of the diabetes–oral-health link; and (iii) healthcare-provider awareness and interprofessional collaboration. The evidence indicated that many patients are unaware of their increased oral-health risk and that oral-health guidance is inconsistently delivered by healthcare professionals however, where guidance was provided, oral-hygiene behaviors and outcomes were improved.

**Conclusions:**

Within the limits of the current study, it could be concluded that oral health is under-addressed in T2DM care and that patient awareness and interprofessional referral pathways are frequently inadequate. These findings support the need for coordinated, evidence-based oral-health promotion within diabetes management and for higher-quality primary studies.

## Introduction

Type 2 diabetes mellitus is associated with several oral health conditions including periodontal disease, dental caries, xerostomia and oral candidiasis (1). Severe periodontitis is among the most prevalent chronic conditions worldwide; the Global Burden of Disease program ranks it among the leading oral disorders by prevalence, and it shares a well-documented bidirectional relationship with diabetes (2, 3). Hyperglycemia contributes to periodontal tissue breakdown, while periodontal inflammation can adversely affect glycemic control, and individuals with poorly controlled diabetes are at particularly high risk of severe periodontal and dental complications (3).

Despite this elevated risk, people with diabetes do not visit dentists frequently. Reported barriers include financial constraints, low perceived need for dental care, discomfort during appointments, and scheduling difficulties, all of which contribute to delayed treatment and higher rates of tooth loss (4). At a systemic level, limited integration between medical and dental services, weak referral pathways and constrained clinician time further reduce access to preventive oral care for this population (5, 6).

Individuals with diabetes often demonstrate limited knowledge of the diabetes–oral-health link, lower awareness of oral-health risk, and do not follow the instructions provided by dentists (5). Even though oral diseases are manageable, there is a lack of clear rationale for coordinated preventive strategies and characterization of current evidence. Because the aim was to map the breadth and nature of a heterogeneous body of evidence rather than to answer a narrowly defined effectiveness question, a scoping-review design was selected. Accordingly, the current scoping review to map the current evidence on oral-health promotion and prevention for adults with type 2 diabetes, including patient awareness, provider practices, barriers to care and preventive strategies.

## Methods

This review was conducted as a scoping review following the framework of Arksey and O'Malley (7), modified by Levac et al. (8) and the JBI methodological guidance (9). Reporting follows the PRISMA extension for Scoping Reviews (PRISMA-ScR) (10). A scoping-review protocol was developed *a priori* to define the objective, eligibility criteria, search strategy and charting approach. The review was not prospectively registered on PROSPERO, which does not accept scoping reviews.

### Research questions

- How prevalent is the lack of oral-health information and dental recommendations provided by healthcare professionals to individuals with type 2 diabetes?
- What barriers prevent people with type 2 diabetes from seeking appropriate oral healthcare despite their heightened risk?
- In what ways can healthcare professionals effectively communicate the increased oral-health susceptibility of people with type 2 diabetes?
- What impact does informative guidance from healthcare professionals, and emphasis on dental consultation, have on the oral-health practices of people with diabetes?
- What strategies can encourage sound oral-hygiene habits among people with type 2 diabetes, and how might these be integrated into routine diabetes management?

### Eligibility criteria (PCC)

Eligibility was formed using the Population–Concept–Context (PCC) guidelines. Population: adults (≥18 years) with type 2 diabetes mellitus. Concept: oral-health promotion and prevention, including patient awareness, provider practices, barriers and preventive strategies. Context: any healthcare setting, any country. Studies were eligible if they were peer-reviewed, published in English between 2015 and 2025, and reported on oral-health prevention or promotion relevant to adults with type 2 diabetes. Editorials, opinion pieces, conference abstracts, and unpublished or pre-print material were excluded, in keeping with the requirement that this article type draw only on published data.

### Information sources and search strategy

Five electronic databases were searched: PubMed, MEDLINE, Web of Science, ProQuest and Google Scholar. Google scholar articles were searched for up to 10 pages for evaluation. The final search was executed in 2025 and covered the period 2015–2025. The search combined controlled vocabulary and free-text terms across two concept blocks using the Boolean operators AND/OR. An illustrative PubMed search string was: (“oral health” OR “dental health” OR “periodontal disease” OR “oral hygiene” OR “dental care”) AND (“diabetes mellitus” OR “type 2 diabetes” OR “T2DM” OR “diabetic”) AND (adult^*^ OR “adults”). The strategy was adapted to the syntax of each database. Reference lists of included studies were hand-searched for additional eligible records.

### Study selection

**Figure 1:**
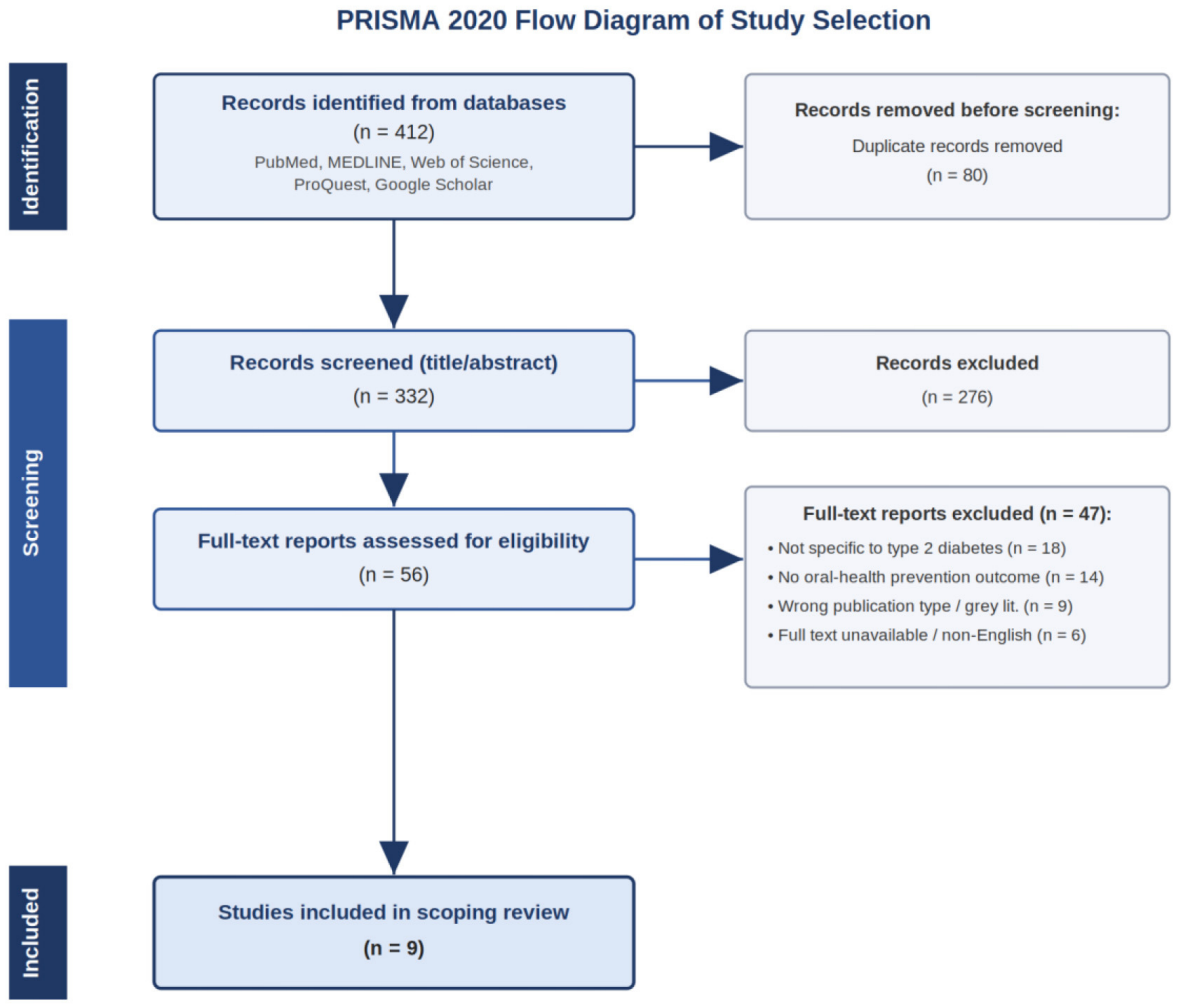
PRISMA 2020 flow diagram of the study-selection process for the scoping review.

All records were imported into a reference manager and de-duplicated. Two reviewers independently screened titles and abstracts against the eligibility criteria, and then independently assessed full texts of potentially relevant records. Disagreements were resolved by discussion and, where necessary, by consultation with a third reviewer. The selection process and the number of records at each stage are summarized in the PRISMA flow diagram (Figure 1).

### Data charting

Data were charted into a piloted extraction form capturing author, year, country, study design, population, aim, and key findings. Charting was performed by one reviewer and verified by another reviewer.

### Critical appraisal

Consistent with scoping-review methodology, formal risk-of-bias assessment was not the primary objective; however, a descriptive appraisal of study design and relative methodological strength was recorded during charting (Table 1) to inform interpretation. The absence of a formal, tool-based quality assessment is acknowledged as a limitation.

**Table 1:** Characteristics and key findings of the nine included studies.

References	Title	Country/Region	Study design	Theme	Key findings
Al-Ansari et al. (11)	Oral hygiene and glycemic control among adults with type 2 diabetes: a cross-sectional study	Saudi Arabia	Cross-sectional (quantitative)	Oral hygiene, glycemic control and outcomes	Adults maintaining good oral hygiene showed better glycemic control and a lower risk of periodontal disease than those with irregular dental care or poor glycemic management
Singh et al. (12)	Oral hygiene and glycemic control in adults with type 2 diabetes: a cross-sectional study	India	Cross-sectional (quantitative)	Oral hygiene, glycemic control and outcomes	Good oral hygiene, regular dental care and controlled blood glucose were associated with a significantly reduced risk of periodontal and other oral diseases
Zhang et al. (13)	Oral hygiene and glycemic control among adults with type 2 diabetes: a cross-sectional study	China	Cross-sectional (quantitative)	Oral hygiene, glycaemic control and outcomes	Regular dental care and good glycaemic control were associated with a significantly lower risk of periodontal disease, suggesting oral hygiene and glucose management may help prevent dental complications
Chen et al. (14)	The relationship between diabetes and oral health: a systematic review	Multi-country	Systematic review	Oral hygiene, glycaemic control and outcomes	Individuals with type 2 diabetes who received regular dental care and maintained good glycaemic control had a significantly lower risk of periodontal and other oral diseases
Siddiqi et al. (15)	Diabetic patients' knowledge of the bidirectional link: are dental health care professionals effectively conveying the message?	Multi-country	Systematic review	Patient knowledge and awareness	73% of diabetic individuals were unaware of the bidirectional link between diabetes and oral health
Poudel et al. (16)	Oral health knowledge, attitudes and practices of people living with diabetes in South Asia: a scoping review	South Asia	Scoping review	Patient knowledge and awareness	People with diabetes generally recognized the importance of dental health and undertook some preventive measures, but lacked complete information and had scope to improve their oral-care routines and attitudes
Siddiqi et al. (17)	Diabetes mellitus and periodontal disease: the call for interprofessional education and collaborative care—a systematic review	Multi-country	Systematic review	Provider awareness and interprofessional care	50% of medical practitioners were aware of oral-health issues and one-third lacked knowledge of the diabetes–oral-health link; only 30% referred patients for oral evaluation, highlighting weak interprofessional collaboration
Poudel et al. (6)	Perceptions and practices of general practitioners on providing oral health care to people with diabetes—a qualitative study	Australia	Qualitative	Provider awareness and interprofessional care	GPs recognized the importance of oral care in diabetes but reported barriers including absent referral pathways, limited training and knowledge, and time constraints
Gambhir et al. (18)	COVID-19: a survey on knowledge, awareness and hygiene practices among dental health professionals	India	Cross-sectional survey	Provider awareness and interprofessional care	Revealed significant gaps in dental practitioners' understanding of key aspects of infection control, illustrating knowledge gaps that can affect care delivery to at-risk groups such as people with diabetes

### Synthesis and consultation

Charted findings were grouped into themes mapped to the five research questions, with quantitative results, summarized descriptively and qualitative findings integrated within the corresponding themes. Given the heterogeneity of designs, populations and outcomes, no meta-analysis was undertaken. In an optional consultation step, preliminary themes were reviewed for relevance and practical utility to inform recommendations for policymakers, practitioners and patients.

## Results

### Study selection and characteristics

The database search identified 412 records. After removal of 80 duplicates, 332 records were screened by title and abstract, of which 276 were excluded. Fifty-six full-text reports were assessed for eligibility and 47 were excluded (reasons in Figure 1), leaving nine studies for inclusion. The study-selection process is presented in Figure 1.

The nine included studies comprised four cross-sectional quantitative studies, three systematic reviews, one qualitative study and one scoping review, conducted across South Asia, East Asia, the Middle East, Australia and multi-country settings (Table 1). Reflecting the descriptive appraisal recorded during charting, most were rated as novel methodological approaches, although outcome measures and instruments varied widely (questionnaires ranged from four to 40 items), and this heterogeneity limited direct comparison. Findings are synthesized below under three themes aligned to the research questions.

*Theme 1—Oral hygiene, glycemic control and oral-health outcomes (RQ4)*

Four studies examined the relationship between oral-hygiene practices, glycemic control and oral-health outcomes (11–14). Across these quantitative and systematic-review sources, adults who maintained good oral hygiene, attended for regular dental care and achieved better glycemic control consistently showed a lower risk of periodontal and other oral diseases. Taken together, these studies indicate a convergent, though observational, association between preventive oral-care behaviors and improved outcomes.

*Theme 2—Patient knowledge and awareness of the diabetes–oral-health link (RQ1, RQ3)*

Two studies characterized patient awareness (15, 16). Siddiqi et al. (15) reported that 73% of people with diabetes were unaware of the link between diabetes and oral health. Poudel et al. (16) found that, while people with diabetes in South Asia generally recognized the importance of dental health and undertook some preventive measures, they lacked complete information and had clear scope to improve their oral-care routines and attitudes.

*Theme 3—Provider awareness, practices and interprofessional collaboration (RQ1, RQ2, RQ5)*

Three studies addressed provider awareness and system-level factors (6, 17, 18). Siddiqi et al. (17) found that only half of medical practitioners were aware of oral-health issues in diabetes, one-third lacked knowledge of the link, and just 30% referred patients for oral evaluation, underscoring weak interprofessional collaboration. Poudel et al. (6) reported that general practitioners recognized the importance of oral care but were constrained by absent referral pathways, limited training and time pressures. Gambhir et al. (18) illustrated broader knowledge gaps among dental professionals that can affect care of at-risk groups.

## Discussion

This scoping review mapped nine peer-reviewed studies on oral-health promotion for adults with type 2 diabetes and identified three inter-related themes. The evidence suggests that oral health is under-addressed in diabetes care, patient awareness of the diabetes–oral-health link is frequently low, and preventive oral-health guidance is inconsistently delivered by healthcare professionals. Where guidance and regular dental attendance did occur, preventive behaviors and oral-health outcomes tended to be better. These observations should, however, be interpreted cautiously given the small number of heterogeneous, largely observational studies.

The finding that many patients are unaware of their elevated oral-health risk (15, 16) suggests that people with diabetes may deprioritize dental care relative to other aspects of their condition (19). The low rates of awareness and referral reported by Siddiqi et al. (17) and the structural barriers described by Poudel et al. (6) recommend stronger interprofessional education and collaborative care. Primary-care clinicians are well-placed to deliver brief risk communication, education and timely dental referral, but require appropriate training, accessible screening tools and functioning referral pathways to do so (20, 21).

Integrating oral health into diabetes management therefore appears to depend on action at three different levels including strengthening patient awareness through targeted education; equipping medical and dental providers with knowledge, tools and clear referral routes; and embedding oral-health promotion within routine diabetes self-management support (4). Dental hygienists and the wider dental team may have a particular role in oral-health screening and promotion, provided that barriers such as limited knowledge of the diabetes–oral-health association and restricted access to evaluation tools are addressed (22). These recommendations follow directly from the themes identified rather than from evidence beyond the included studies and are offered as directions warranting further evaluation rather than as established practice.

The included studies varied in design (cross-sectional, qualitative, systematic and scoping reviews), population, geographical setting and outcome measurement, and several relied on self-reported data. This variability, together with the small number of eligible studies, limits comparability and precludes any pooled estimate; it also means the review maps the shape of the evidence rather than quantifying effects. Much of the evidence originates from higher-resource or specific regional settings, which may reflect limited research capacity for oral-health–diabetes integration in lower-resource contexts and constrains generalizability.

### Strengths and limitations

This review applied an established scoping-review framework, used a multi-database search, employed independent dual screening, and reported findings in line with PRISMA-ScR, synthesizing results transparently against pre-specified research questions. Several limitations should be acknowledged. Consistent with scoping-review methodology, no formal tool-based risk-of-bias assessment was undertaken, so the certainty of individual findings was not graded. The included studies were heterogeneous in design, population and outcome measurement and several used self-reported data, limiting comparability and generalizability. Fourth, the protocol was not prospectively registered. These limitations mean the findings are best regarded as an exploratory map of the current evidence rather than a definitive synthesis.

## Conclusions

The current scoping review indicates that oral health remains under-emphasized in the care of adults with type 2 diabetes. Patient awareness of the diabetes–oral-health link is low, and oral-health guidance and referral are inconsistently provided by healthcare professionals, even though people with diabetes are at the risk of periodontal and other oral diseases. The mapped evidence suggests that coordinated, interprofessional approaches combining patient education, provider training and clear referral pathways could support better oral-health promotion within diabetes management. Given the methodological limitations and the small number of included studies, these conclusions are exploratory and should be tested in higher-quality primary research; they nonetheless reinforce practice-guideline recommendations to incorporate oral health into routine diabetes care.

## Data Availability

The datasets used and/or analyzed during the current study are available from the corresponding author on reasonable request.
