# Correction: Oral health promotion in adults with type 2 diabetes–a scoping review

**DOI:** 10.3389/fmed.2026.1956810

**Published:** 2026-08-14

**Authors:** Frontiers Production Office

**Affiliations:** Frontiers Media SA, Lausanne, Switzerland

**Keywords:** access to dental care, oral health promotion, patient education, periodontal disease, scoping review, type 2 diabetes mellitus

Affiliation “Dental Department, Prince Sultan Military College of Health Sciences, Dhahran, Saudi Arabia” was erroneously included in the paper for author “Yahya Alholimie”. The updated affiliation list appears below:

^1^Dental Department, Prince Saud Bin Jalawy Hospital, Al Mubarraz, Saudi Arabia

^2^Dental and Oral Health Department, Prince Sultan Military College of Health Sciences, Dhahran, Saudi Arabia

^3^Dental Department, King Faisal General Hospital, Al Hofuf, Saudi Arabia

^4^University of Doha for Science & Technology, Doha, Qatar

^5^Jordan University of Science and Technology, Irbid, Jordan

The original version of this article has been updated.

