# Supplementary material for: Oral health promotion in adults with type 2 diabetes–a scoping review
Source: Front Med (Lausanne). 2026 Jul 29;13:1852350. doi: 10.3389/fmed.2026.1852350 (PMC13461489; doi:10.3389/fmed.2026.1852350)
Supplement: Supplementary file 1 [file Table_1.docx]

**ORAL HEALTH PROMOTION IN ADULTS WITH DIABETES TYPE II:**

**A SCOPING REVIEW**

**Table 1: Resources/Studies Included in this Review.**

| **Resources/Studies Included in this Study** | | | | | |
| --- | --- | --- | --- | --- | --- |
| **Authors** | **Title** | **Year** | **Quality** | **Type of Study** | **Findings** |
| Al-Ansari et al. | Oral hygiene and glycemic control among adults with type 2 diabetes: A cross-sectional study | 2020 | High Quality | Cross-sectional quantitative study | Individuals maintaining good oral hygiene exhibit better glycemic control and lower risk of periodontal disease compared to those with irregular dental care or poor glycemic management. |
| Chen et al. | The relationship between diabetes and oral health: a systematic review | 2019 | High Quality | Systematic Review | Individuals with type 2 diabetes who received regular dental care and maintained good glycemic control had a significantly lower risk of periodontal and other oral diseases. |
| Gambhir et al. | Covid-19: a survey on knowledge, awareness and hygiene practices among dental health professionals in an Indian scenario | 2020 | Moderate Quality | Quantitative survey | The study's results revealed significant gaps in dental practitioners' understanding of critical parts of COVID-19. |
| Siddiqi et al. | Diabetes mellitus and periodontal disease: The call for interprofessional education and interprofessional collaborative care - A systematic review of the literature | 2022 | High Quality | Systematic Review | The results showed that 50% of medical practitioners were aware of oral health issues, while one-third lacked knowledge about the link between diabetes mellitus and oral health. Only 30% referred their patients for oral health evaluations. The findings also highlighted a lack of interprofessional collaboration between medical and dental practitioners in managing diabetic patients. |
| Siddiqi et al. | Diabetic patients' knowledge of the bidirectional link: are dental health care professionals effectively conveying the message? | 2019 | High Quality | Systematic Review | The review found that 73% of diabetic individuals were unaware of the link between diabetes and oral health. |
| Singh et al. | Oral hygiene and glycemic control in adults with type 2 diabetes: a cross-sectional study | 2018 | High Quality | Cross-sectional quantitative study | Individuals with type 2 diabetes who maintained good oral hygiene, received regular dental care, and had controlled blood glucose levels showed a significantly reduced risk of periodontal and other oral diseases. |
| Poudel et al., | Oral Health Knowledge, Attitudes and Practices of People Living with Diabetes in South Asia: A Scoping Review | 2022 | High Quality | Systematic Review | The review found that diabetic individuals in South Asian nations generally understood the importance of good dental health and engaged in certain preventative measures. However, they lacked complete information and had an opportunity to enhance their oral health care routines and attitudes. |
| Poudel et al., | Perceptions and practices of general practitioners on providing oral health care to people with diabetes - a qualitative study | 2020 | High Quality | Qualitative Study | The findings show that many GPs understand the significance of oral healthcare for diabetic individuals, acknowledging their compromised immune capacity and are at greater risk of infection. The barriers identified in the research included referral pathways absence, limited training and knowledge, and time limitations |
| Zhang et al. | Oral hygiene and glycemic control among adults with type 2 diabetes: a cross-sectional study | 2017 | High Quality | Cross-sectional quantitative study | The results indicate that individuals with type 2 diabetes who received regular dental care and maintained good glycemic control had a significantly lower risk of developing periodontal disease. The findings suggest that optimal oral hygiene and blood sugar management may prevent dental complications in this population. |
